# Supplementary material for: MCProj: metacell projection for interpretable and quantitative use of transcriptional atlases
Source: Genome Biol. 2023 Oct 5;24:220. doi: 10.1186/s13059-023-03069-7 (PMC10552220; doi:10.1186/s13059-023-03069-7)
Supplement: Supplementary file 1 — Additional file 1: Fig S1. Validation: leave-one-batch-out projections. Similar to Fig. 2A. Counting cells classified according to their reference annotated type and the type assigned to their query metacell by scARCHES. Fig S2. Validation: leave-one-batch-out projections. Similar to Fig. 2A. Counting cells classified according to their reference annotated type and the type assigned to their query metacell by Seurat. Fig S3. Validation: leave-one-batch-out projections. Similar to Fig. 2A. Counting cells classified according to their reference annotated type and the type assigned to their query metacell by scMAP. Fig S4. Validation: leave-one-type-out projections. Similar to Fig. 3A. Counting cells classified according to their reference annotated type and the type assigned to their query metacell by scARCHES. Fig S5. Validation: leave-one-type-out projections. Similar to Fig. 3A. Counting cells classified according to their reference annotated type and the type assigned to their query metacell by Seurat. Fig S6. Validation: leave-one-type-out projections. Similar to Fig. 3A. Counting cells classified according to their reference annotated type and the type assigned to their query metacell by scMAP. Fig S7. The fraction of query cells for which a synthetic technology difference was applied (and was not corrected), which were either not successfully projected, or which were assigned a type different than the expected type, for each of the methods we compared, very close to Fig. 2B. [file 13059_2023_3069_MOESM1_ESM.pdf]

SUPPLEMENTARY FIGURE S1

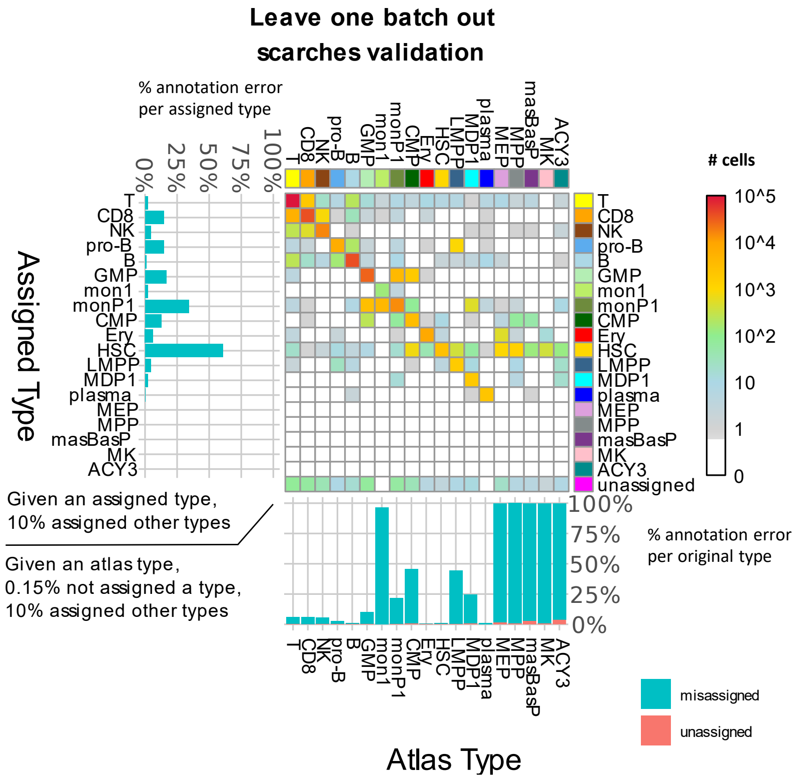

Fig S1: Validation: leave-one-batch-out projections. Similar to Fig2A.

Counting cells classified according to their reference annotated type and the type assigned to their query metacell by scARCHES.

SUPPLEMENTARY FIGURE S2

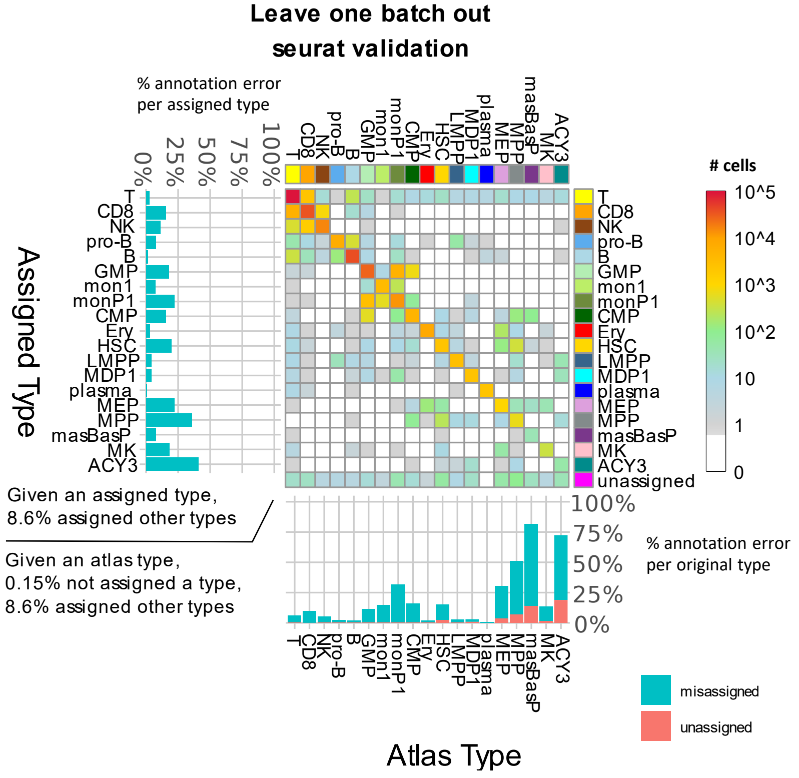

Fig S2: Validation: leave-one-batch-out projections. Similar to Fig2A.

Counting cells classified according to their reference annotated type and the type assigned to their query metacell by Seurat.

SUPPLEMENTARY FIGURE S3

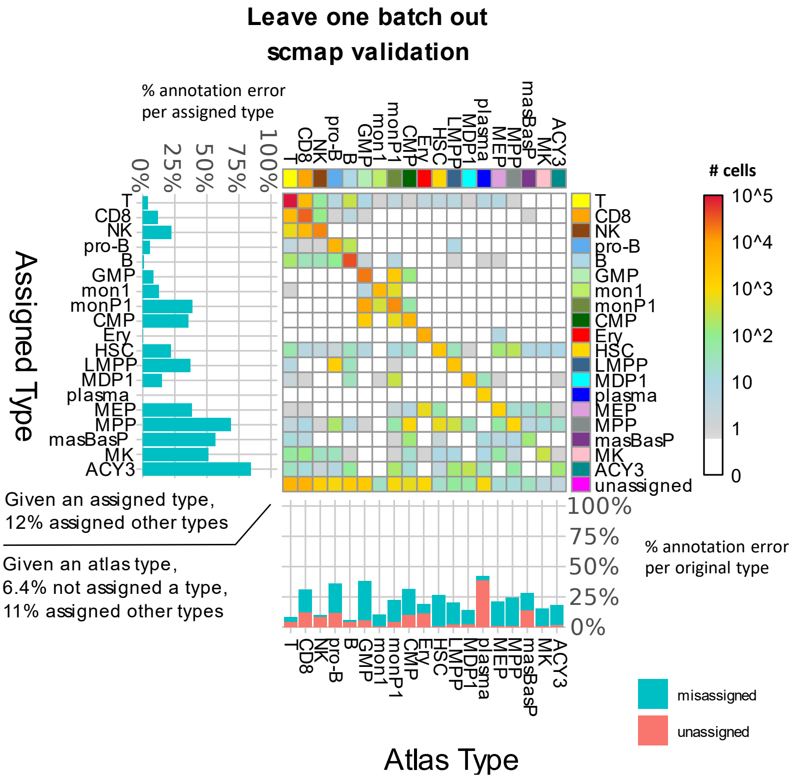

Fig S3: Validation: leave-one-batch-out projections. Similar to Fig2A.

Counting cells classified according to their reference annotated type and the type assigned to their query metacell by scMAP.

SUPPLEMENTARY FIGURE S4

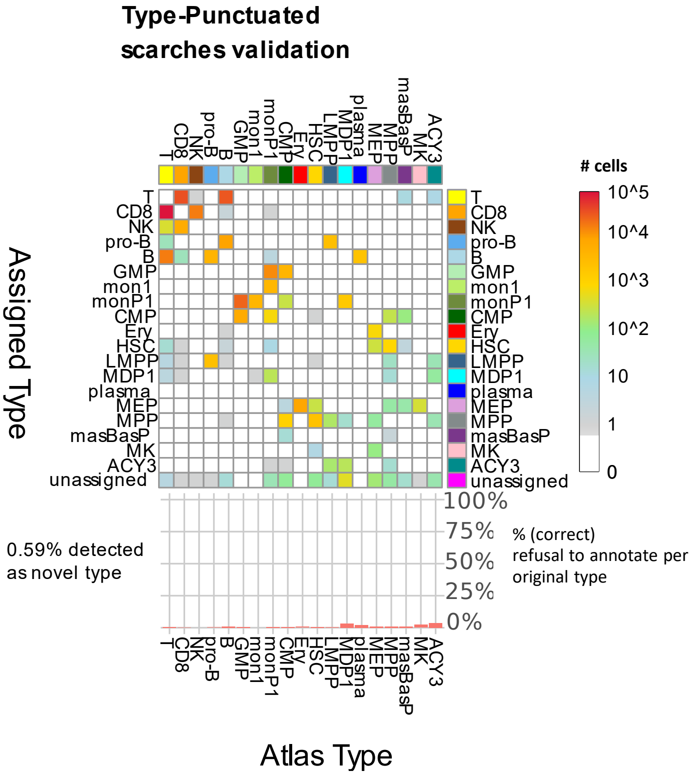

Fig S4: Validation: leave-one-type-out projections. Similar to Fig 3A.

Counting cells classified according to their reference annotated type and the type assigned to their query metacell by scARCHES.

SUPPLEMENTARY FIGURE S5

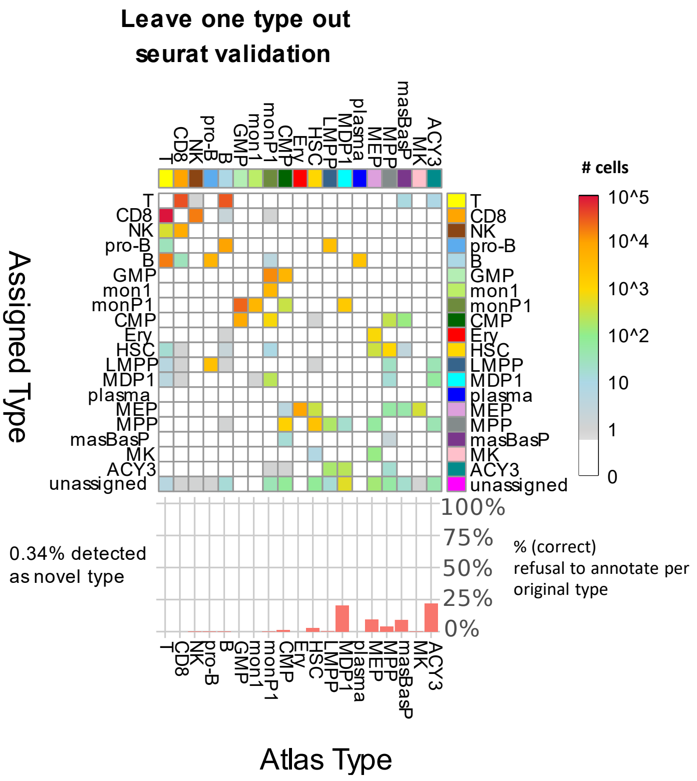

Fig S5: Validation: leave-one-type-out projections. Similar to Fig 3A.

Counting cells classified according to their reference annotated type and the type assigned to their query metacell by Seurat.

SUPPLEMENTARY FIGURE S6

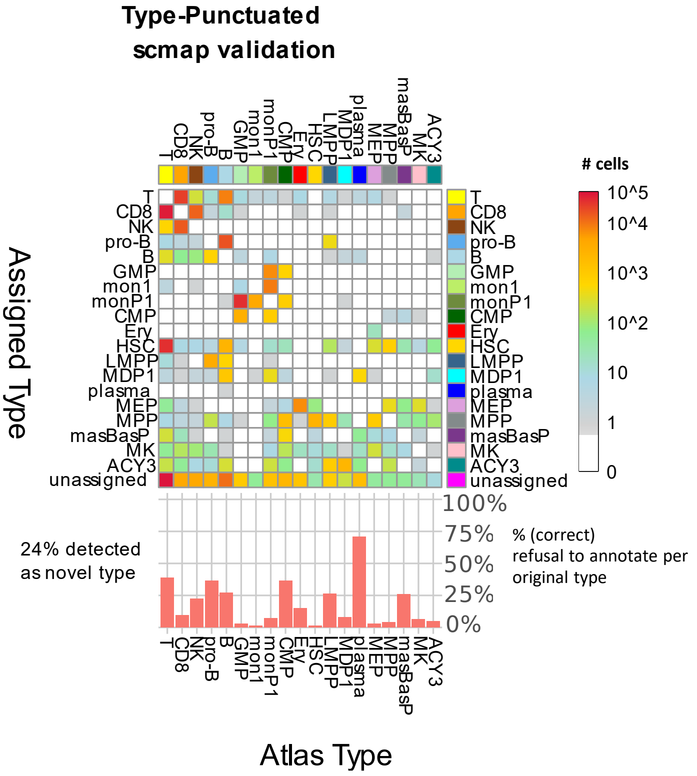

Fig S6: Validation: leave-one-type-out projections. Similar to Fig 3A.

Counting cells classified according to their reference annotated type and the type assigned to their query metacell by scMAP.

SUPPLEMENTARY FIGURE S7

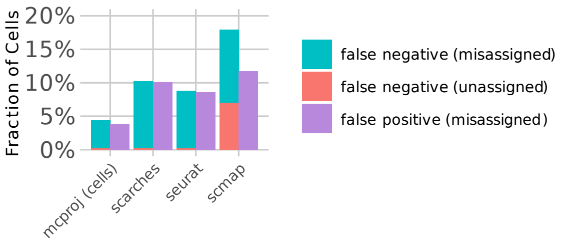

Fig S7: The fraction of query cells for which a synthetic technology difference was applied (and was not corrected), which were either not successfully projected, or which were assigned a type different than the expected type, for each of the methods we compared, very close to Fig 2B.
